# Mapping the Landscape of Electronic Health Records and Health Information Exchange Through Bibliometric Analysis and Visualization

**DOI:** 10.7759/cureus.59128

**Published:** 2024-04-27

**Authors:** Jeena Joseph, Anat Suman Jose, Gilu G Ettaniyil, Jasimudeen S, Jobin Jose

**Affiliations:** 1 Department of Computer Applications, Marian College Kuttikkanam (Autonomous), Kuttikkanam, IND; 2 Department of Library, St. Peter's College Kolenchery, Kolenchery, IND; 3 Department of Library, St. Thomas College of Teacher Education, Pala, IND; 4 Department of Library, St. Stephen's College Uzhavoor, Uzhavoor, IND; 5 Department of Library, Marian College Kuttikkanam (Autonomous), Kuttikkanam, IND

**Keywords:** vosviewer., biblioshiny, bibliometric analysis, health information exchange, electronic health records

## Abstract

The adoption of Electronic Health Records (EHRs) and the establishment of Health Information Exchange (HIE) systems have significantly transformed healthcare delivery and management. This study presents a comprehensive bibliometric analysis and visualization of the landscape surrounding EHRs and HIE to provide insights into the current state and emerging trends in this field. Leveraging advanced bibliometric methodologies, including co-citation analysis, keyword co-occurrence analysis, and network visualization techniques, we systematically map the scholarly literature spanning several decades. Our analysis reveals key thematic clusters, influential publications, prolific authors, and collaborative networks within the domain of EHRs and HIE. Furthermore, we identify significant research gaps and areas for future exploration, including interoperability challenges, privacy concerns, and the integration of emerging technologies such as artificial intelligence and blockchain. The findings of this study offer valuable insights for researchers, policymakers, and healthcare practitioners seeking to navigate and contribute to the ongoing evolution of EHRs and HIE systems, ultimately enhancing the quality, efficiency, and accessibility of healthcare services.

## Introduction and background

In modern healthcare systems, Electronic Health Records (EHRs) and Health Information Exchange (HIE) are critical components. The Health Information Technology for Economic and Clinical Health (HITECH) Act of 2009 boosted hospital use of Electronic Health Records (EHR) [1]. EHRs contain multifaceted, diverse, multimodal, infrequent data and time series, such as laboratory test results, doctor's written notes, patient prescriptions, demographic details, diagnoses, epidemiological studies, behavioral data, etc. Clinical responsibilities based on this enormous data set can range from acute care to long-range planning. Also, information from electronic health records can aid in therapy selection, identifying patient similarities, incorporating genetic data for customized treatment, forecasting hospital length of stay (LoS), and predicting patients' readmission risks [2].

The HIE is an essential element of the Health Information Technology (HIT) architecture designed to ease the electronic transfer of patient information between medical institutions throughout the treatment process [3]. HIE can increase the effectiveness, safety, and quality of healthcare services. Despite their potential benefits, EHRs and HIE are accompanied by technological limitations, monetary constraints, a lack of compatibility, and security and privacy challenges, which are the most frequently mentioned hurdles [4]. In addition, there are a variety of healthcare stakeholders who might be both vendors and consumers of health information. Coordination among all parties involved might be complex due to legal, security, confidentiality, and operational concerns [5,6]. Healthcare patients are essential stakeholders since their agreement is required to share their personal medical information. Patients' views about revealing confidential medical data may impact the design of forthcoming health information systems [5,6].

Artificial intelligence (AI) methods have been widely utilized in numerous domains of the ‎healthcare field, encompassing personalized treatment plans, offering evidence-based ‎recommendations derived from analyses of patient data, error identification, and accurate ‎disease diagnosis [7]. Additionally, AI can play a pivotal role in EHR systems, smoothing the path for exchange ‎and synergy of health data in diverse healthcare environments, which is vital for integrated ‎care coordination. However, given the AI's limitations, exercising utmost care and caution ‎becomes vital when embracing an AI chatbot in this context [8]. Moreover, it is essential to identify and use a suitable tool that can effectively help ‎standardize the evaluation process for assessing the accuracy and reliability of health ‎information generated by AI-based models. Ensuring the responsible and cautious ‎implementation of AI technologies in healthcare systems is essential, ultimately delivering ‎benefits to patients and healthcare providers alike [9].

Bibliometric analysis is a computer-based method utilized in scientific research to quantitatively evaluate publications within a specific field or subject matter [10-12]. This technique not only identifies key authors and core research but also elucidates the connections between them through the statistical analysis of books, journals, and other publications. Essential for tracking an author's output and influence, bibliometric analysis is also instrumental in determining journal impact factors and visualizing publication linkages to better understand scholarly networks [13-15]. Serving multiple purposes, it pinpoints seminal works and core literature, monitors the evolution of research trends, and assesses the impact of individual researchers and institutions. Widely applied in science and technology studies, library and information science, and research policy-making, bibliometric analysis supports strategic planning and funding decisions. The methodology encompasses data collection from bibliographic databases like Web of Science, Scopus, or Google Scholar, followed by data cleaning to eradicate duplicates and rectify inaccuracies, and comprehensive data analysis [16,17]. This analysis may include mapping scientific fields, performing citation analysis to trace influence and knowledge flows, and conducting network analysis to explore relationships among authors, institutions, and countries. Techniques such as co-citation and co-authorship analysis are frequently employed to identify research clusters and collaborative patterns [18]. Overall, bibliometric methods provide an empirical foundation for comprehending the dynamics of scientific research, offering crucial insights for scholars, policymakers, and librarians alike, thereby enabling stakeholders to effectively and strategically navigate the vast landscapes of scientific output [19-22].

RStudio is a software program that provides an integrated environment for working with R, a programming language used for statistical computing and graphics. RStudio comes in two flavors: RStudio Desktop, a standard desktop application, and RStudio Server, which runs on a remote server and can be accessed via a web-based browser [23]. Bibliometrix is a free and open-source application for visually representing scientific literature. It was written in R to use with other analytical and graphical applications [24]. As a result, Bibliometrix has become extremely user-friendly, even for individuals with no programming experience. Bibliometrix contains routines for importing bibliographic information from SCOPUS, Clarivate Analytics' Web of Science, PubMed, Digital Science Dimensions, as well as Cochrane databases, performing bibliometric analysis and generating data matrices for co-citation, association, collaborative research analysis, and co-word analysis. Users can input bibliographic data in a wide range of formats, including BibTeX, RIS, and EndNote XML, and conduct various studies, such as network analysis, bibliometric analysis, and keyword mining. The application provides multiple visualization options, such as network schematics, word clouds, and heat maps, to help users better understand their data and communicate the results they explore [23,25]. It is an indispensable tool for scholars, librarians, and other specialists who work with bibliographic information because it helps them to obtain different perspectives and make well-informed decisions based on their findings.

VOSviewer, a versatile and user-friendly software tool, plays a pivotal role in visualizing and analyzing bibliometric data, facilitating a deeper understanding of scholarly landscapes. Leveraging innovative algorithms, VOSviewer enables researchers to construct and visualize co-authorship networks, co-citation networks, and keyword co-occurrence networks with remarkable clarity and precision. Its intuitive interface empowers users to explore complex relationships among documents, authors, and keywords, thereby uncovering hidden patterns and trends within vast bibliographic databases [26,27]. Moreover, VOSviewer offers customizable visualization options, allowing users to tailor visual representations to their specific research objectives. With its robust features and user-centric design, VOSviewer continues to serve as an indispensable tool for bibliometric analysis, contributing to the advancement of scientific inquiry across diverse disciplines.

The study's research objectives in the field of Electronic Health Records (EHR) and Health Information Exchange (HIE) encompass a comprehensive analysis spanning from 2005 to 2024. The goals include assessing trends in scholarly output to identify and evaluate the publication dynamics over the years, including periods of growth and decline. It also aims to identify key publications and journals by analyzing the volume of documents they have published, which will highlight the most prolific sources in the field. Another significant objective is to evaluate the impact of individual researchers by examining the extent of their contributions and their association with specific research topics. This study will also map the research landscape through keyword co-occurrence analysis, which will uncover central themes, technological focuses, and socio-economic considerations within the literature. Additionally, it intends to analyze international collaboration by studying the network of co-authorship globally to understand collaboration patterns and the roles different countries play. Lastly, the research will explore interdisciplinary connections to pinpoint potential areas for innovative research and development across different research areas within the field.

The scientific literature for this study was systematically sourced from the Scopus database as of April 7, 2024. The search terms "Electronic Health Records" AND "Health Information Exchange" were employed to capture relevant documents published between January 2005 and April 2024, without language restrictions. Inclusion criteria were set to encompass articles, conference papers, and book chapters to ensure a comprehensive analysis, focusing on documents that explicitly discuss or evaluate EHRs or HIE. Exclusion criteria involved omitting letters, reviews, and surveys to concentrate on original research, as well as any documents missing essential bibliographic elements such as author names or titles. The retrieved records were stored in CSV files containing full records and cited references, which were analyzed using biblioshiny and VOSviewer tools. This process identified 896 relevant papers, and the results were detailed in Table 1, which includes the duration of publication, sources used, average citations per document, total authors, and other relevant metrics, thus providing comprehensive insights into the literature surveyed.

**Table 1:** Overall information about articles on EHRs and HIE during 2005-2024 EHR: Electronic Health Record; HIE: Health Information Exchange

Main Information about Data	
Timespan	2005:2024
Sources (Journals, Books, etc.)	336
Documents	896
Annual Growth Rate %	6.82
Document Average Age	7.25
Average citations per doc	17.45
References	23513
Document Contents	
Keywords Plus (ID)	3622
Author's Keywords (DE)	1658
Authors	
Authors	3085
Authors of single-authored docs	74
Authors Collaboration	
Single-authored docs	84
Co-Authors per Doc	4.61
International co-authorships %	12.39
Document Types	
article	693
book chapter	54
conference paper	149

## Review

Annual scientific production

**Figure 1:**
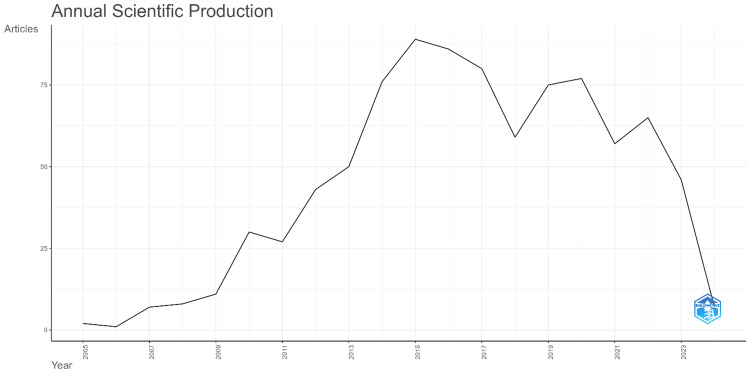
The annual scientific production from 2005 to 2024 generated using biblioshiny

Figure 1 represents the number of articles published per year in the field of Electronic Health Records and Health Information Exchange from the year 2005 to 2023. Starting from 2005, there's a visible initial period of low production with fewer than 25 articles per year. This is followed by a steady increase in the number of articles until a sharp uptick beginning around 2009, leading to a peak of approximately 100 articles around 2013. Following this peak, there is a slight fluctuation but with a general maintenance of high productivity until about 2019. After 2019, there is a marked and precipitous decline, culminating in a sharp drop to the lowest point on the graph in 2023, where the number of articles published appears to be less than 25, roughly equal to the counts from the initial years. This drastic decrease might indicate a significant shift in the field, reduced interest, or perhaps external factors affecting scientific output in this domain.

Most relevant sources

**Figure 2:**
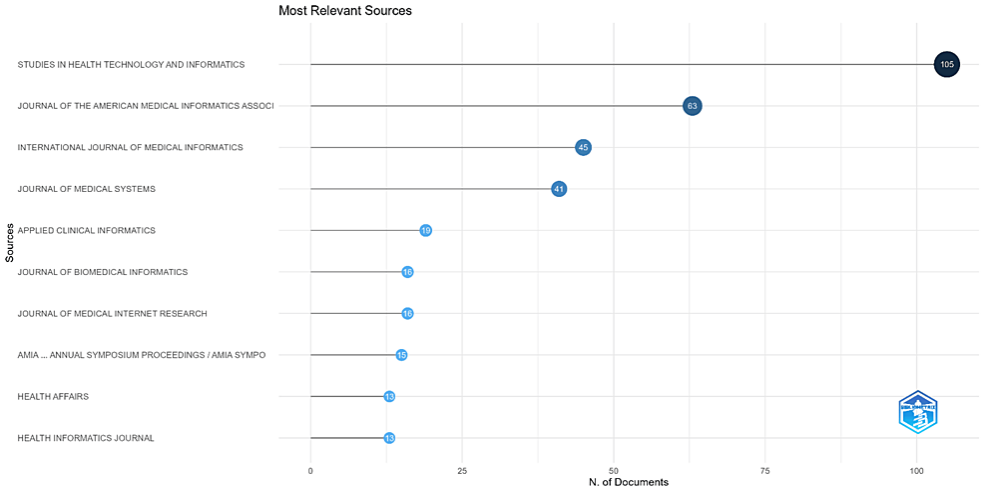
The most relevant sources generated using biblioshiny

Figure 2 displays the number of documents published by various sources in the domain of Electronic Health Records and Health Information Exchange. The sources are listed on the vertical axis, and the horizontal axis represents the number of documents, indicated by the position and size of the bubbles. The largest bubble is associated with "Studies in Health Technology and Informatics," which has published 105 documents, making it the most prolific source. The second largest is the "Journal of the American Medical Informatics Association" with 63 documents. Following these, the "International Journal of Medical Informatics" has contributed 45 documents, and the "Journal of Medical Systems" has produced 41. Other sources such as "Applied Clinical Informatics," "Journal of Biomedical Informatics," and "Journal of Medical Internet Research" have contributed between 15 to 19 documents each. Finally, the "AMIA... Annual Symposium Proceedings / AMIA Symposium," "Health Affairs," and "Health Informatics Journal" are the sources with the fewest documents, each with 13. This chart visually communicates the relative contribution of each source to the field, with the size of each bubble indicating the significance of the source's role in the dissemination of research and information regarding Electronic Health Records and Health Information Exchange.

Most relevant authors

Table 2 lists relevant authors alongside the number of articles they have published. Adler-Milstein J. is the most prolific author with 35 articles, indicating a significant contribution to the field. Following close behind is Dixon BE with 30 articles and Vest JR with 28 articles, both of whom also represent substantial scholarly output. Kaushal R has contributed 27 articles, suggesting a strong presence in the academic discourse. Finally, Patel V has authored 17 articles, which, while less than the others mentioned, still signifies a noteworthy level of engagement with research in this area. This distribution of articles among authors can provide insights into the key contributors and thought leaders within the domain of Electronic Health Records and Health Information Exchange.

**Table 2:** Top five authors ranked by number of articles

Authors	Articles
Adler-Milstein J	35
Dixon BE	30
Vest JR	28
Kaushal R	27
Patel V	17

Three-field plot

**Figure 3:**
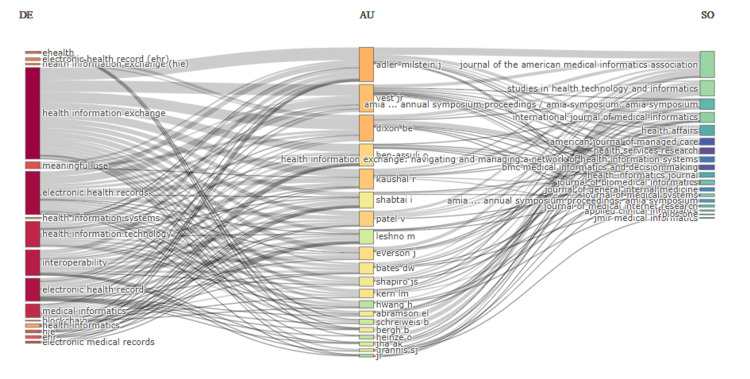
Three-field plot visualizing the relationship between keywords (DE), authors (AU), and sources (SO) generated using biblioshiny

The three-field plot in Figure 3 is used to visualize the relationship between keywords (DE), authors (AU), and sources (SO) of scientific articles. In this plot, the left column (DE) displays keywords from the articles, which are various terms relevant to the field of study in this case, terms like "eHealth," "electronic health record (EHR)," and "health information exchange (HIE)." These terms are likely topics or subjects addressed in the body of literature. The center column (AU) lists authors who have contributed to the field, with names like Adler-Milstein J., Dixon BE, Vest JR, Kaushal R, and Patel V, among others. These are the same authors mentioned in the previous message, indicating their prominence in the research community. The right column (SO) lists the sources of publication, such as journals and proceedings, including the "Journal of the American Medical Informatics Association," "Studies in Health Technology and Informatics," and "Health Affairs." These sources are where the research on the topics listed has been published. The lines connecting the columns illustrate the association between specific keywords, authors, and sources. A line that connects the keyword "electronic health records" with Adler-Milstein J. in the center and then with the "Journal of the American Medical Informatics Association" on the right, would suggest that Adler-Milstein J. has published articles on electronic health records in that journal. This visualization helps to quickly identify which authors are the most prolific in certain areas of research and which sources publish the majority of the work in these topics.

Co-occurrence of keywords

**Figure 4:**
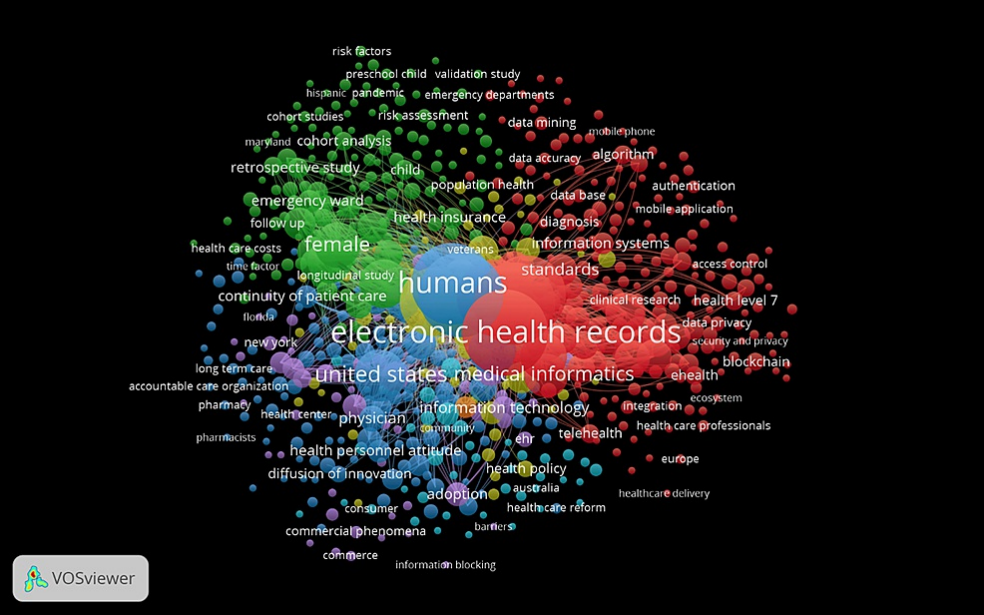
Co-occurrence of keywords generated using VOSviewer

Figure 4 is a keyword co-occurrence network visualization generated by VOSviewer. This type of visualization maps out the relationships between various terms used in a set of scientific publications. The most prominent keyword, "electronic health records," sits at the center of the visualization, suggesting it is a core topic with a high degree of connectivity to other terms. It is surrounded by other significant keywords such as "medical informatics," "information systems," and "information technology," which are likely closely related topics in the literature. The keywords are depicted in different colors, which typically represent clusters or groups of keywords that are often mentioned together in the literature, indicating related subfields or contexts. The Red Cluster encapsulates the technical and security dimensions of EHRs, with a concentration on safeguarding digital health information. This is evidenced by recurrent keywords such as "information systems," "standards," "access control," "data privacy," "security and privacy," "blockchain," and "authentication," which collectively highlight the significant focus on developing robust technical standards and exploring cutting-edge technologies such as blockchain for enhancing the security of health data management systems. Adjacent to this, the Green Cluster surfaces, addressing the socio-economic and demographic ramifications of EHRs. The presence of terms like "female," "veterans," "health insurance," and "health care costs" indicates an array of studies probing into how EHRs intersect with societal and economic factors, their influence on specific demographic groups, and the subsequent implications for insurance frameworks and healthcare expenditures. Meanwhile, the Blue Cluster gravitates toward the policy-oriented and geographical narratives of EHR implementation. Keywords such as "United States," "adoption," "health policy," "diffusion of innovation," "barriers," alongside state-specific mentions like "Florida" and "New York," signify an investigative trend into how EHRs are being integrated across different locales, what political and practical challenges are encountered, and how policy can facilitate or hinder the progress of EHR adoption. Finally, the Light Blue Cluster sheds light on the clinical utilization and healthcare provision aspects of EHRs. With keywords such as "longitudinal study," "continuity of patient care," "long term care," and "accountable care organization," this cluster underscores research dedicated to understanding the longitudinal impacts of EHRs, their role in ensuring continuity of care, and the broader implications for organized healthcare delivery systems. Each cluster, delineated by its unique color and thematic focus, contributes a piece to the complex puzzle of EHR research, reflecting the multifaceted nature of this technological advancement in modern healthcare.

Co-authorship between countries

**Figure 5:**
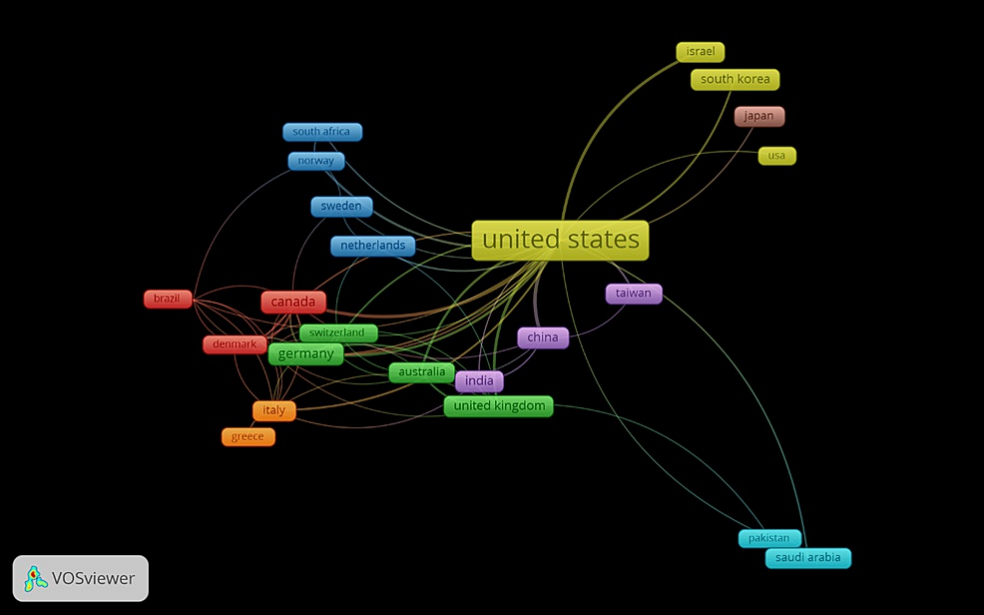
Co-authorship of countries generated using VOSviewer

Figure 5 is a network of co-authorship between countries in the field of Electronic Health Records and Health Information Exchange. The visualization highlights the United States as the central and most prominent node, which suggests it has the highest number of collaborations internationally within this dataset. This central position, combined with the numerous lines extending to various other countries, indicates that researchers in the United States are extensively involved in international co-authorship. Countries are represented by nodes of varying sizes and colors, and the lines between them depict the collaborations. The thickness of the lines signifies the volume of co-authored papers, suggesting that thicker lines represent stronger or more frequent collaborations. The colors represent different clusters or groups of countries that frequently collaborate with each other, indicating regional or thematic commonalities. For instance, there are visible clusters among European countries like Germany, Italy, and the Netherlands, which are connected closely to each other and also to the United States, indicating robust cross-Atlantic research partnerships. Asian countries such as China, Japan, and South Korea form another cluster with strong ties to the United States, suggesting significant collaborative research activities between these nations. Less prominent nodes like Brazil and India are still connected to the United States and others, indicating their participation in international research efforts, albeit with fewer co-authored works than the central nodes. The overall network demonstrates a global landscape of research where the United States stands out as a hub of scholarly collaboration, connecting various international communities and facilitating a diverse and interconnected research environment.

Discussion

The study provides a comprehensive overview of the research landscape in the field of Electronic Health Records and Health Information Exchange from multiple perspectives. Starting with the temporal dynamics of scholarly output, it reveals a clear growth pattern in the number of articles published from 2005 to around 2019, reflecting the burgeoning interest and development within the field. The peak around 2013 could be associated with technological advancements and increased funding for healthcare IT initiatives. However, the subsequent decline, particularly the sharp drop in 2023, could be indicative of a market reaching maturity, shifts in research funding, changes in regulatory focus, or the culmination of initial research enthusiasm. It also raises questions about the future trajectory of the field and the factors influencing these trends.

The analysis of the most relevant sources highlights the journals and proceedings that are central to the dissemination of research in this domain. "Studies in Health Technology and Informatics" and the "Journal of the American Medical Informatics Association" stand out as leading publications. The distribution of sources may guide researchers in choosing where to publish and which sources to consult for the latest findings in the field. The identification of key authors underlines the significant contributions of individuals like Adler-Milstein J. and Dixon BE. These authors not only advance the field through their scholarly work but also likely play a role in shaping research agendas and guiding younger researchers. Three-field plot underscores the interconnectedness of specific research topics, key authors, and publication sources, illustrating a cohesive network of scholarly communication. This provides valuable insights into the most influential areas of research and the experts who are at the forefront of these topics.

The keyword co-occurrence visualization offers a multi-faceted view of the field's thematic structure. The clustering of keywords into distinct themes suggests areas of concentrated research effort, such as technical aspects of EHRs, socio-economic impacts, policy and implementation challenges, and clinical applications. Each cluster presents a nuanced understanding of the field's complexity and the interplay between technology, policy, and healthcare delivery. Finally, the co-authorship network highlights the role of international collaboration in the field. The centrality of the United States suggests its dominance in research output and its integral role in fostering international research partnerships. The visualization also reveals regional collaborations and potential gaps where international partnerships could be strengthened.

There are several critical research gaps in the realm of EHRs and HIE systems. Interoperability challenges persist as a significant barrier, preventing seamless data sharing and communication across different platforms and systems. Privacy concerns are paramount, with current systems lacking robust mechanisms to safeguard against breaches and unauthorized access. Additionally, the integration of emerging technologies such as artificial intelligence and blockchain remains underexplored, indicating potential areas for impactful research and development.

Emerging trends and future explorations identified in the study suggest promising directions for advancing EHR and HIE capabilities. The adoption of AI and machine learning could revolutionize predictive analytics and patient care personalization. Blockchain technology offers potential for enhancing security and transparency in data exchanges. Furthermore, the development of advanced data analytics tools is crucial for extracting valuable insights from vast amounts of health data, which could significantly improve public health monitoring and individual care strategies.

Limitations of the study

The reliance on Scopus databases might exclude important grey literature or non-academic reports, limiting the breadth of analyzed material. Additionally, there is a technological bias that may undervalue simpler, yet effective solutions in favor of more complex technologies, possibly skewing the findings towards high-tech approaches. These limitations underscore the need for a cautious interpretation of the results and suggest areas for methodological improvement in future studies.

## Conclusions

The bibliometric analysis offers a detailed snapshot of the research landscape in Electronic Health Records and Health Information Exchange over nearly two decades. The study highlights a period of rapid growth and high productivity in the field, followed by a recent decline that calls for careful consideration of the underlying causes. The analysis has revealed key journals and authors who have significantly influenced the field, providing a roadmap for upcoming researchers to navigate the literature and establish collaborative networks. The thematic clusters identified reflect the broad spectrum of research interests, ranging from technical security concerns to socio-economic factors, policy issues, and clinical applications. These themes suggest targeted areas for future research and development. The visualization of international co-authorship emphasizes the value of cross-border collaboration in driving innovation and addressing global health challenges. The practical implications of this study are relevant to a diverse set of stakeholders, including academics, healthcare professionals, policymakers, and industry leaders, highlighting the importance of staying current with research trends, fostering collaborative environments, and strategically investing in areas that could further enrich the domain of health informatics. This study not only maps the historical trajectory of research productivity but also sets the stage for future explorations and interventions to sustain the growth and impact of this critical field.
